# Effects of the ketogenic diet on skin—potential benefits and risks

**DOI:** 10.3389/fnut.2025.1686056

**Published:** 2025-10-16

**Authors:** Natalia Chylińska, Mateusz Maciejczyk

**Affiliations:** ^1^Independent Laboratory of Cosmetology, Medical University of Białystok, Bialystok, Poland; ^2^Department of Hygiene, Epidemiology and Ergonomics, Medical University of Białystok, Bialystok, Poland

**Keywords:** ketogenic diet, skin, acne, psoriasis, hidradenitis suppurativa, skin cancer

## Abstract

In recent years, interest in the impact of diet on the skin has increased significantly, and dietary interventions are now considered an essential part of managing certain dermatological conditions. Among them, the ketogenic diet (KD) has gained particular attention. KD is a high-fat, very low-carbohydrate, and moderate-protein dietary approach that induces ketosis, defined by serum ketone body concentrations exceeding 0.5 mmol/L. Although initially developed for the treatment of drug-resistant epilepsy in children, its applications have broadened over time. Current evidence on KD and the skin remains limited, focusing mainly on inflammatory skin diseases such as acne, psoriasis, and hidradenitis suppurativa (HS), as well as cutaneous melanoma. In this review, we summarize the existing data on these conditions and discuss the molecular mechanisms underlying the potential effects of KD, including anti-inflammatory and antioxidant pathways, modulation of signaling cascades, and interactions with the gut microbiota. Finally, we also address the reported adverse effects of KD on the skin.

## 1 Introduction

Numerous scientific studies have confirmed the role of diet in maintaining the proper functioning of the human body and in the prevention and treatment of various diseases, including obesity, diabetes, and cardiovascular disease (1–3). As the level of education in society increases, so too does awareness of the impact of diet on the skin (4). Dietary factors can have both positive and adverse effects on the skin (5). A balanced and varied diet provides the skin with adequate nutrients, which play a key role in maintaining the integrity of the skin barrier and promoting skin health (6). Proper nutrition improves hydration and elasticity and delays skin aging (7). An unhealthy diet plays a key role in the development of many skin conditions, including inflammatory skin diseases (8). Certain dietary patterns, particularly those diets high in saturated fatty acids (SFAs), simple sugars, and highly processed foods, may exacerbate inflammation in conditions such as acne vulgaris, psoriasis, atopic dermatitis, and hidradenitis suppurativa (HS) (5, 9, 10). Some dietary factors may also contribute to premature skin aging and the development of skin cancer (11, 12). Dietary interventions are currently considered an important element in the treatment of skin conditions. They can be used as a preventive measure or adjuvant therapy for chronic skin conditions (11, 12). Several dietary patterns, including the Mediterranean diet (MD), intermittent fasting, the ketogenic diet (KD), and gluten-free and vegan diets, are recognized for their skin-benefiting effects. These diets differ primarily in their macronutrient composition (13). Among these, KD is gaining popularity due to promising results in the treatment of various skin diseases (13, 14). KD is a low-carbohydrate dietary regimen that has gained widespread public recognition, particularly in the treatment of drug-resistant epilepsy in children and rapid fat reduction (15, 16). Widely promoted in the media, it has become one of the most commonly used weight loss diets worldwide (16). Although its mechanisms of action are not fully understood, KD's contemporary applications extend beyond its original use (17). Recent reports indicate that KD may also have a potential therapeutic role in supporting the treatment of selected skin conditions, most notably inflammatory dermatoses such as acne, psoriasis, hidradenitis suppurativa, as well as cutaneous melanoma (14, 18–21). As the available evidence is still limited, this review focuses on these diseases while placing particular emphasis on the molecular mechanisms—such as modulation of inflammatory pathways, promotion of weight loss, strengthening of antioxidant defense against reactive oxygen species (ROS), and alterations in the gut microbiota—which may provide broader insights into the potential effects of KD on skin health (14, 18–20).

## 2 History of the ketogenic diet

The earliest reports on the use of a low-carbohydrate diet to reduce the severity of symptoms, especially epileptic seizures, date back to antiquity (22). Hippocrates claimed that the body of an epilepsy patient should be purified. He used fasting similar to KD for this purpose (22–25). These early ancient practices laid the foundations for the development of the modern KD pattern (23, 25, 26). Nevertheless, the core concept of KD emerged in the 1860s (26). Entrepreneur William Banting pioneered the low-carbohydrate diet (27). Banting, who struggled with obesity and related complications, limited his carbohydrate intake on the advice of Dr. William Harvey (27). The first references to this diet appeared in medical literature in 1864 (28). However, this model differed significantly from contemporary KD standards. The proposed weight loss diet excluded specific carbohydrate products, such as bread, butter, milk, potatoes, and sugar (28). In 1869, William Banting published the fourth edition of his book, “Letter on Corpulence,” which proclaimed the high effectiveness of a low-carbohydrate diet in reducing body fat (29). The low-carbohydrate diet gained wide public acceptance (28, 29).

In 1911, French doctors Guelpe and Marie published the first scientific report on the benefits of intermittent fasting in epilepsy (30). Another breakthrough in the popularization of KD came in 1921 (16). Dr. Rollin Woodyatt, an American endocrinologist, observed that starvation caused blood glucose levels to decrease and acetone and β-hydroxybutyric acid levels to increase (16, 31, 32). At the same time, another American physician, Russell Wilder, concluded that a low-carbohydrate diet was a more beneficial alternative to radical fasting. He recommended a low-carbohydrate diet as a treatment for epileptic seizures, especially in children (16, 32–34). Wilder also introduced the term “ketogenic diet” into general use, referring to a diet high in fat and low in carbohydrates (28).

In 1925, Dr. Mynie Gustav Peterman, a pediatrician and Wilder's colleague, developed the classic KD, which consisted of 1 g of protein per kilogram of the child's body weight, 10–15 g of carbohydrates per day, with fats making up the remaining energy requirements (31, 35). Peterman confirmed the effectiveness of KD in the treatment of epileptic seizures in children (31, 36).

Advances in the pharmaceutical industry and technologies, particularly the discovery of phenytoin in 1938, laid the foundation for the development of effective anti-seizure medications. Drug therapy replaced the use of KD over the following decades (28, 37, 38). KD enjoyed a resurgence in popularity in the 1990s (39). In 1994, Dateline National Broadcasting Company (NBC) television aired a report on the case of Charlie Abrahams, the son of American film director and screenwriter Jim Abrahams. The boy, who had epilepsy, was treated with KD, which successfully reduced his epileptic seizures (39, 40). KD has once again been found to have application in the treatment of chronic neurological diseases (37).

## 3 Ketogenic diet

The World Health Organization (WHO) recommends that adults limit their total fat intake to less than 30% of their daily energy intake (41). In the diets of children aged 2 years and over and of adults, fats should mainly be derived from unsaturated fatty acids (41). It is recommended that saturated fats account for no more than 10% of daily energy intake and trans fats for no more than 1% (41). It is also recommended that free sugars be limited to less than 10% of total energy intake and that adults consume vegetables and fruits of at least 400 g/day, as well as naturally occurring fiber of at least 25 g/day (41).

KD significantly deviates from standard healthy nutrition recommendations. KD is a dietary model primarily based on high-fat foods, combined with a minimal intake of carbohydrates (42, 43). Compared to other low-carbohydrate diets, KD is characterized by the strictest carbohydrate restriction (42). Overall, the diet is predominantly fat-based, with a moderate proportion of protein and only a small fraction of carbohydrates (44). The goal of this diet is to induce ketosis. Ketosis is a metabolic state in which the concentration of ketone bodies exceeds 0.5 mmol/L (42, 45). The upper limit of ketosis is considered to be 3 mmol/L (42, 45, 46).

Several common variants of KD exist, differing in the macronutrient ratios (47, 48). These include the standard ketogenic diet (SKD), the targeted ketogenic diet (TKD), the cyclical ketogenic diet (CKD), and the high-protein ketogenic diet (HPKD). The different variations of KD are selected to suit the individual patient's needs and therapeutic goals (Table 1) (47–49).

**Table 1:** Characteristics of the ketogenic diet types.

**Type of ketogenic diet**	**Nutritional composition**	**Goal and benefits**	**References**
Standard ketogenic diet (SKD)	The most common type of ketogenic diet; it typically consists of about 70–75% fat, 20–25% protein, and 5–10% carbohydrates	Most commonly recommended for the treatment of epilepsy and weight loss	(47, 48, 259)
Targeted ketogenic diet (TKD)	Provides additional carbohydrates during exercise	Recommended for physically active individuals	(32, 47, 49)
Cyclical ketogenic diet (CKD)	Involves periods of higher carbohydrate intake with periods of low carbohydrate intake	Recommended for individuals who engage in intensive exercise; it increases energy levels during training	(47, 49, 260)
High-protein ketogenic diet (HPKD)	Involves a higher protein intake, usually consisting of 60% fat, 35% protein, and 5% carbohydrates	Recommended for athletes to reduce body weight while maintaining muscle mass	(47, 260)

Other KD types are also used for therapeutic purposes, such as the very low energy ketogenic therapy (VLEKT), formerly referred to as very low-calorie ketogenic diet (VLCKD), the modified Atkins diet (MAD), the medium-chain triglyceride ketogenic diet (MCTKD), and the low glycemic index treatment (LGIT) (50, 51). These dietary approaches share the principles of the traditional KD but differ in total energy supply, macronutrient distribution, and degree of restrictiveness. MAD, MCTKD, and LGIT are generally less restrictive, more palatable, and easier to follow in the long term (52, 53). VLEKT represents a medically supervised intervention primarily recommended for patients with obesity and metabolic or inflammatory conditions (54).

The VLEKT therapy was introduced by the “KetoNut” expert panel of the Italian Society of Nutraceuticals (SINut) and the Italian Association of Dietetics and Clinical Nutrition (ADI). VLEKT is characterized by a very low total energy intake (650–800 kcal per day), carbohydrate restriction to less than 30 g/day, fat intake of around 20 g/day with a preference for sources of unsaturated fats such as extra-virgin olive oil, and protein intake of 0.8–1.2 g/kg of reference body weight (i.e., calculated based on the patient's height) (54). The updated nomenclature was introduced to reduce confusion in the literature and to emphasize the therapeutic framework of this intervention, rather than merely its caloric restriction (54).

MAD was developed in 2003 as an alternative to the conventional KD, particularly in the context of treating drug-resistant epilepsy (55). It recommends a carbohydrate intake of 10–20 g/day (48, 55). MAD does not require limiting calories, fluids, or protein (56). In MAD, the ratio of fats to carbohydrates and proteins is 1:1 or 2:1 (51).

MCTKD was developed in 1971 by Huttenlocher et al. (57, 58). In MCTKD, medium-chain triglycerides (MCTs) are digested and metabolized much faster than long-chain triglycerides (LCTs) (59). MCTs enter the liver directly via the portal vein and are rapidly converted to ketones (59). In MCTKD, MCTs provide up to 60% of total energy intake. Consequently, MCTKD is easier to adhere to and maintain long-term than the classic KD (59). The primary natural sources of MCTs are coconut oil, which contains high amounts of lauric acid (C12:0), and palm kernel oil, which includes caprylic acid (C8:0) and capric acid (C10:0) (59, 60). Literature reports indicate that the use of MCTKD is not without adverse reactions. Gastrointestinal side effects, such as vomiting, diarrhea, or bloating, are often reported during MCTKD use (51, 58).

LGIT recommends consuming less than 50 g, or 40–60 g, of low glycemic index (GI) carbohydrates per day, which accounts for 10% of total energy intake (36, 52, 53, 61). In LGIT, proteins account for about 20–30% and fats for 60% of daily energy intake (53, 61). This KD variety prevents blood glucose spikes after a meal, which may reduce the risk of epileptic seizures (36).

KD is not a universal diet; therefore, to achieve health benefits, the patient's individual needs must be considered (62). KD can affect lipid levels, electrolytes, and renal and hepatic function (63). Thus, it should be introduced under close medical supervision, taking into account food allergies and intolerances (62, 64). It is common practice, especially in children with drug-resistant epilepsy, to introduce KD in a hospital setting (64). The classic hospital KD protocol involves a 24–48-h fast to induce metabolic ketosis, after which the KD ratio is gradually increased, starting with a 1:1 ratio (1 g of fat per 1 g of protein and carbohydrates), until a 4:1 ratio is achieved (61, 64, 65). Once KD has been fully implemented, the patient continues the diet in an outpatient setting (52). Hospitalization is particularly recommended in cases requiring intensive caregiver training, monitoring of epileptic status, and strict metabolic control in infants (65). Another proposed option does not require hospitalization, and the KD ratio is increased weekly, starting at 1:1, until a 4:1 ratio is achieved (61, 65). Forgoing fasting in this option improves dietary tolerance, reducing the acute side effects of rapid ketosis (61, 65). The International Ketogenic Diet Study Group (IKDSG) recommends thorough laboratory diagnosis before and during KD to ensure optimal safety and effectiveness (52, 62, 66). The laboratory tests include a complete blood count with platelet count, blood glucose levels, electrolytes (potassium, sodium, and chloride), liver and kidney function tests (albumin, creatinine, and blood urea nitrogen), fasting lipid profile, vitamin D levels, a serum acylcarnitine profile, and a urinalysis (52, 62, 66). In the case of epilepsy, it is additionally recommended to perform electroencephalography (EEG) and 3 Tesla (3T) magnetic resonance imaging (MRI) of the head. Patients with suspected heart disease are additionally recommended to have an echocardiogram (ECG) (52, 67).

According to IKDSG guidelines, KD use is strictly contraindicated in patients with a deficiency of carnitine (primary), carnitine palmitoyltransferase (CPT) 1 or 2, carnitine translocase, pyruvate carboxylase, medium-chain acyl-coenzyme A (CoA) dehydrogenase (MCAD), long-chain acyl-CoA dehydrogenase (LCAD), short-chain acyl-CoA dehydrogenase (SCAD), and disorders of fatty acid β-oxidation and porphyria. Further, KD should not be used in patients with chronic kidney disease, liver failure, hypertriglyceridemia-induced acute pancreatitis, heart failure, especially NYHA Class IV, eating disorders, respiratory failure, as well as in the elderly, and during pregnancy and lactation (52, 62, 63, 67, 68).

**Figure 1:**
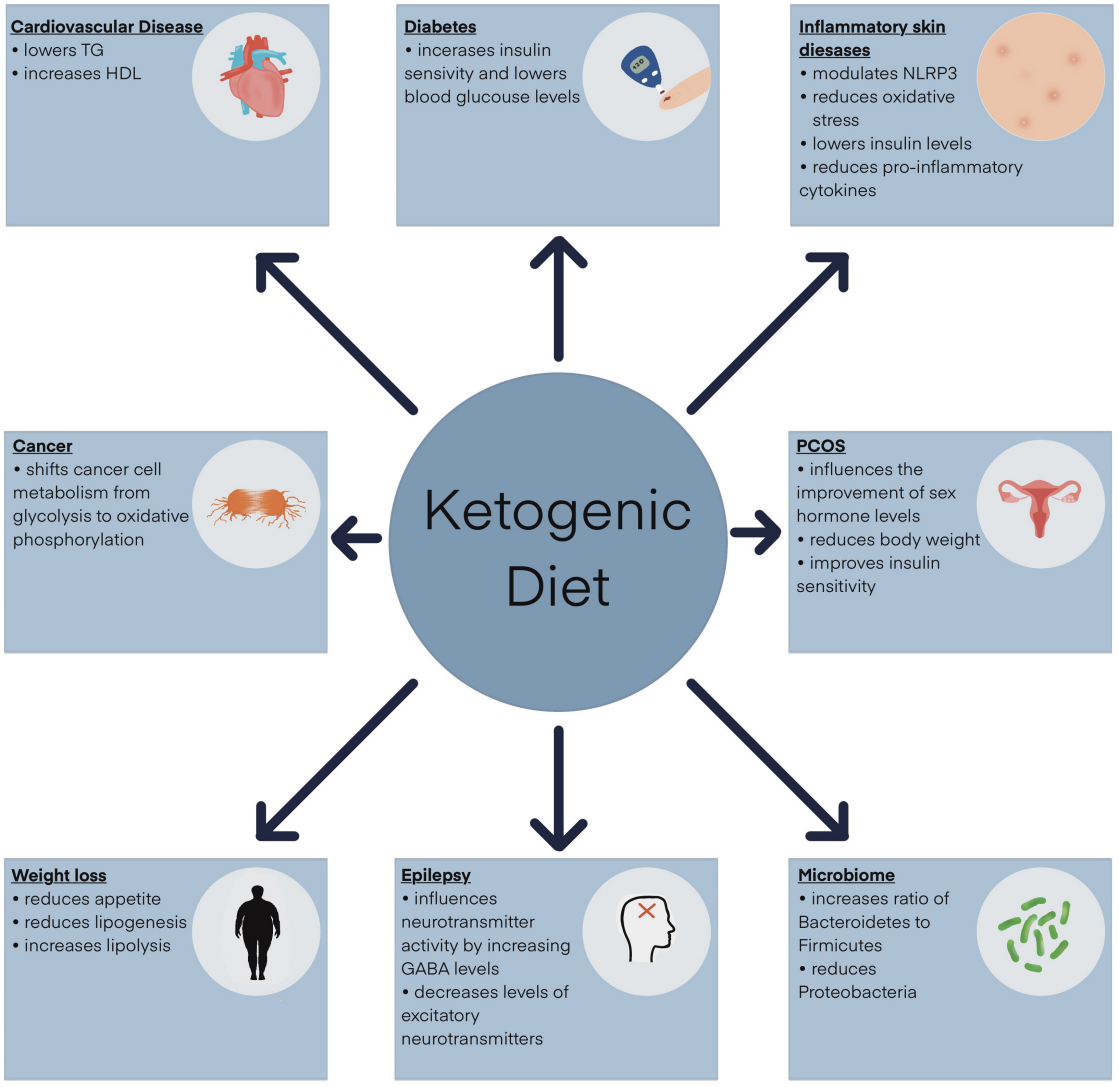
Clinical application of the ketogenic diet. GABA, γ-aminobutyric acid; HDL, high-density lipoprotein; NLRP3, NOD-like receptor pyrin domain-containing protein 3 inflammasome; TG, triglycerides.

KD is widely used as an adjuvant therapy for many diseases. It is a well-established therapeutic approach for the treatment of drug-resistant epilepsy, particularly in children. Research findings indicate that after 3 months of KD use, the frequency of epileptic seizures decreases by at least 50% (69, 70). It has also been applied successfully in patients with overweight and obesity (71). Meta-analyses of clinical trials indicate that KD leads to faster weight loss in the short term, with optimal results usually achieved within the first 3–6 months (71, 72). A key aspect of KD compared to other weight loss diets is its ability to regulate hunger and satiety (71, 73). KD has also emerged as a promising dietary intervention for treating insulin resistance and type 2 diabetes (74). Scientific evidence shows that it reduces fasting glucose and glycated hemoglobin (HbA1c) levels in patients with type 2 diabetes (75). KD also shows potential in the prevention and treatment of cardiovascular disease (76). This diet usually leads to an increase in HDL cholesterol and a decrease in triglycerides (76, 77). However, there is evidence that it may also increase LDL cholesterol in some patients, which increases the risk of cardiovascular disease. KD has also shown promising effects on Alzheimer's disease (AD) and Parkinson's disease (PD) (78). Studies suggest that KD may support cognitive function in early AD and motor function in PD patients (78). There is preliminary evidence that it may benefit individuals with autism spectrum disorder (ASD) (79). Numerous scientific studies also confirm the beneficial effects of KD in treating certain types of cancer (39, 80, 81). The key anti-cancer mechanism of KD is carbohydrate reduction, which leads to lower glucose levels and slower proliferation of cancer cells (39, 82). KD is also used in glucose transporter 1 (GLUT1) deficiency syndrome to provide an alternative source of energy for the brain in conditions of impaired glucose access (83). It is also a beneficial dietary intervention in polycystic ovary syndrome (PCOS) (84). In addition, KD's effect on reducing oxidative stress and inflammation has also been confirmed (85). The role of KD in reducing the severity of symptoms in many skin conditions and its beneficial effects on the skin are also gaining recognition (14, 18–20). The therapeutic potential of KD is shown in Figure 1, whereas the key mechanisms specifically related to skin health are summarized in Table 2.

**Table 2:** Molecular mechanisms by which the ketogenic diet (KD) may influence skin health.

**Mechanism**	**Potential effects on the skin**	**References**
Antioxidant effects	Activation of Nrf2 and AMPK pathways; increased antioxidant/detoxifying enzymes; modulation of NAD^+^/NADH balance and SIRT activity; protection against ROS-induced skin damage (studied mainly in acne, psoriasis, melanoma, HS)	(19, 20, 124, 125, 141)
Anti-inflammatory effects	Suppression of NLRP3 inflammasome; reduction of pro-inflammatory cytokines (IL-1β, IL-6, IL-17, IL-18, IL-23); attenuation of inflammatory mediators (studied mainly in acne, psoriasis, hidradenitis suppurativa)	(18, 19, 21)
Regulation of signaling pathways	Modulation of AKT/mTOR, FOXO1, SREBP1, and NF-κB, as well as activation of AMPK (studied mainly in acne, psoriasis, and cutaneous melanoma)	(18, 19, 21)
Gut microbiota modulation	Restoration of gut–skin axis balance; reduction of dysbiosis-associated inflammation and barrier dysfunction (studied mainly in acne, psoriasis, and hidradenitis suppurativa)	(21, 158, 163, 165, 214, 218, 261)
Metabolic effects	Improved insulin sensitivity; reduction of IGF-1 and insulin signaling; increased IGFBP-3 and SHBG; inhibition of SREBP-1–mediated lipogenesis; weight loss benefits (notably in psoriasis and HS); alterations in lipid metabolism, including increased sphingomyelins (cutaneous melanoma)	(19, 21, 222)

AMPK, adenosine 5′-monophosphate-activated protein kinase; AKT, protein kinase B; FOXO1, forkhead box protein O1; HS, hidradenitis suppurativa; IGF-1, insulin-like growth factor; IGFBP-3, insulin-like growth factor-binding protein 3; IL-1β, interleukin-1β; IL-6, interleukin 6; IL-17, interleukin 17; IL-18, interleukin 18; IL-23, interleukin 23; mTOR, mechanistic target of rapamycin; NAD^+^/NADH, nicotinamide adenine dinucleotide (oxidized/reduced form); NF-κB, nuclear factor kappa B; NLRP3, NOD-like receptor pyrin domain-containing protein 3; Nrf2, nuclear factor erythroid 2-related factor 2; ROS, reactive oxygen species; SHBG, sex hormone-binding globulin; SIRT, sirtuin; SREBP1, sterol regulatory element-binding transcription factor 1.

## 4 Ketogenesis and ketone bodies

The proper functioning of the human body is dependent on the energy provided by food (86). Carbohydrates are the primary, but not the only, source of energy necessary for the proper functioning of the brain, central nervous system, and red blood cells (87, 88). The energy substrate with a key role in the human body is glucose (89). Increased blood glucose concentration induces an increase in the production of a polypeptide hormone called insulin (90, 91). Its main task is to regulate glucose to optimal levels for the body (90, 92). Insufficient carbohydrate intake of less than 50 g/day leads to reduced insulin secretion (71, 93). The human body's inability to extract energy from carbohydrates prompts it to use alternative sources of energy (93). The initial stage involves an increase in the concentration of glucagon, a hormone produced by the pancreas (94). Glucagon stimulates the release of glucose from glycogen stores (95, 96). Once these are depleted, the enzymatic process of gluconeogenesis begins. The primary role of this process is to convert amino acids, glycerol, and lactic acid into glucose (96). Nevertheless, gluconeogenesis can only last for a total of 3 days in the human body. At this point, the body's stored fat tissue becomes the next energy source, initiating ketogenesis (35, 97). The breakdown of fatty acids in the liver leads to an increase in the production of ketone bodies. Ketone bodies serve as an alternative energy source for various tissues and organs in the human body. Naturally, the human body continuously produces a small amount of ketone bodies, which can then be used to produce energy (98). However, elevated ketone levels are observed in patients with type 1 diabetes, as well as during intense physical exercise, alcohol abuse, or very restrictive diets (99). Depending on their concentration, ketone bodies can have both beneficial and harmful effects. Their elevated concentration, usually exceeding 7 mmol/L, is observed during fasting. However, a concentration exceeding 25 mmol/L are often found in patients with type 1 diabetes (100). When insulin deficiency is severe, blood glucose levels rise, leading to increased ketone body production. This condition, known as diabetic ketoacidosis, can lead to hazardous complications that are harmful to the patient's health and may even be life-threatening (101). The most serious of these are sepsis and cerebral edema, which can lead to shock and coma (102). There is also a distinction between alcoholic ketoacidosis associated with chronic alcohol abuse and starvation ketoacidosis resulting from a starvation diet (99, 103).

Ketones are a class of organic compounds containing a carbonyl group (C=O) connected to two hydrocarbon groups (R). These molecules are highly soluble in water, so their transport to various tissues does not require the involvement of lipoproteins (104, 105). Ketone bodies in the human body include acetone, acetoacetate (AcAc), and β-hydroxybutyrate (BHB). They have not only metabolic but also signaling functions. All of the compounds mentioned above are formed during ketogenesis (104).

**Figure 2:**
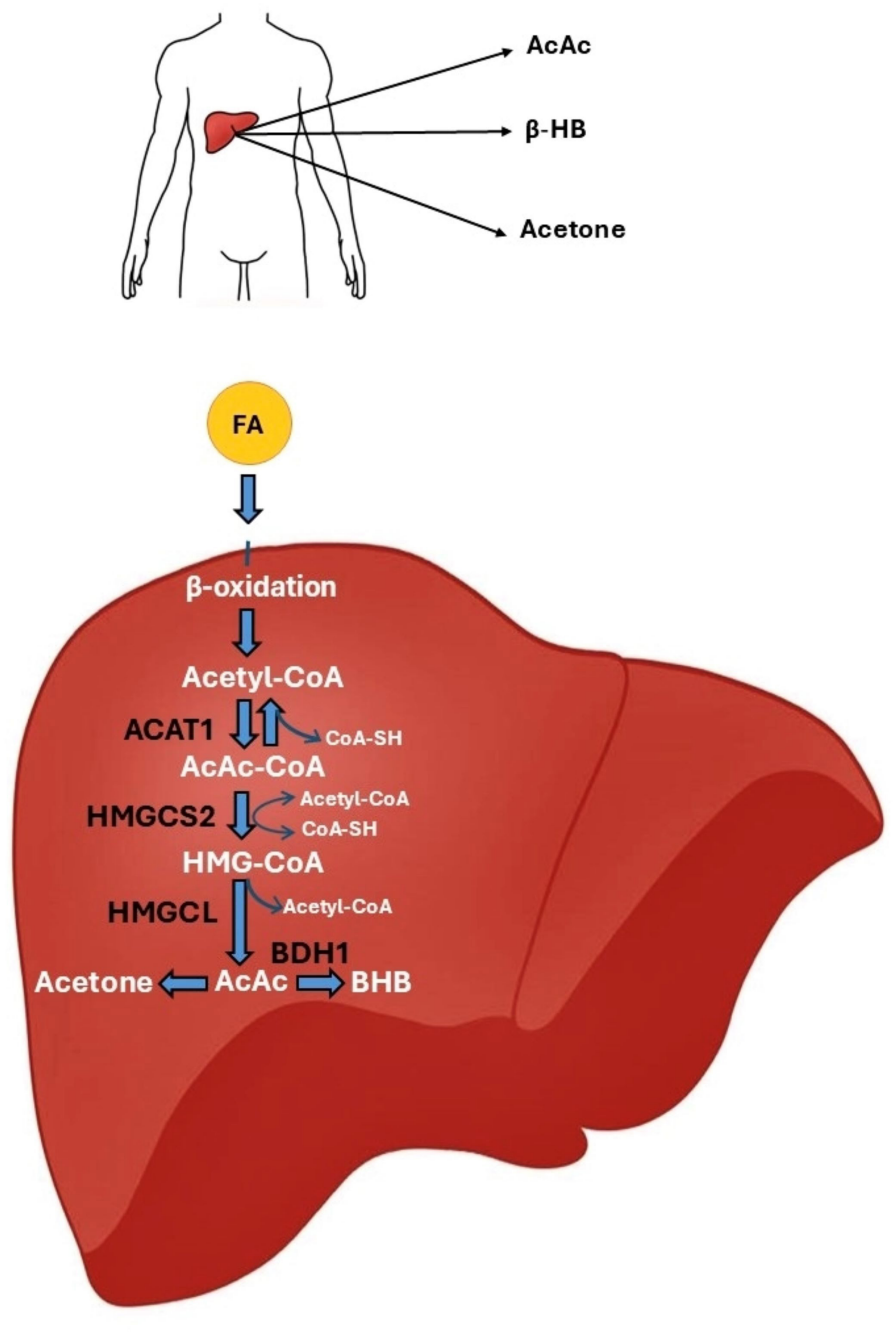
The ketone body synthesis pathway. In the mitochondria of hepatocytes, fatty acids undergo β-oxidation, which converts them into acetyl-CoA molecules. The action of the ACAT1 enzyme results in the fusion of two acetyl-CoA molecules to form AcAc-CoA. AcAc-CoA combines with another acetyl-CoA molecule under the action of the HMGCS2 enzyme to form HMG-CoA. The HMGCL enzyme breaks down HMG-CoA into AcAc. AcAc can be converted to BHB by the BDH1 enzyme or decarboxylated to acetone. AcAc, acetylacetone; ACAT1, acetyl-CoA acetyltransferase 1; BDH1, D-β-hydroxybutyrate dehydrogenase; BHB, β-hydroxybutyrate; HMGCL, 3-hydroxy-methylglutaryl-CoA lyase; HMGCS2, 3-hydroxy-3-methylglutaryl-CoA synthase 2.

Ketogenesis occurs in the liver, specifically, in hepatocytes, through the mitochondrial β-oxidation of fatty acids. It is a complex mechanism. It involves the conversion of acetyl-CoA to ketone bodies: AcAc, BHB, and acetone. In the first step of ketogenesis, the two acetyl-CoA molecules react with each other. Under the influence of thiolase (acetyl-CoA acetyltransferase 1, ACAT1), CoA-SH is detached from one molecule of acetyl-CoA, and acetoacetyl-CoA (AcAc-CoA) is formed. AcAc-CoA then combines with another acetyl-CoA molecule to form 3-hydroxy-3-methylglutaryl-CoA (HMG-CoA). This reaction is catalyzed by 3-hydroxy-3-methylglutaryl-CoA synthase 2 (HMGCS2). Subsequently, 3-hydroxy-methylglutaryl-CoA lyase (HMGCL) cleaves HMG-CoA in an irreversible reaction into acetyl-CoA and the first ketone body, AcAc. AcAc is converted in a reversible reaction catalyzed by D-β-hydroxybutyrate dehydrogenase (BDH1), leading to the formation of BHB, or undergoes non-enzymatic decarboxylation with the formation of acetone (Figure 2) (104, 105).

## 5 Antioxidant potential of the ketogenic diet

The skin is the largest organ of the human body, one that is particularly susceptible to oxidative stress because of its constant exposure to external factors (106, 107). Oxidative stress is defined as an imbalance between the production of ROS and the antioxidant capacity of the body (106). ROS are highly reactive molecules such as hydrogen peroxide (H_2_O_2_), superoxide anion radical (${\text{O}}_{2}^{-}$), hydroxyl radical (OH), and singlet oxygen (^1^O2) (108). Antioxidants are a group of chemical compounds that neutralize ROS (109). Antioxidants can be divided into endogenous antioxidants, such as antioxidant enzymes: superoxide dismutase (SOD), catalase (CAT), and glutathione peroxidase (GPx), as well as exogenous antioxidants, which mainly include non-enzymatic antioxidants such as vitamin C, vitamin E, carotenoids, ubiquinone, and flavonoids (110). An imbalance between oxidation and antioxidant protection damages cellular structures like proteins, lipids, and DNA, which impairs their function (111, 112). Several studies have shown that oxidative stress is involved in the pathogenesis of inflammatory skin diseases, such as atopic dermatitis, acne, allergic dermatitis, and psoriasis (113). It also contributes to accelerated skin aging and can lead to the development of skin cancer (114).

Many factors are known to induce oxidative stress in the skin. These include environmental factors, such as ultraviolet (UV) radiation and environmental pollutants, including ozone and cigarette smoke (115, 116). Other oxidative stress triggers include a poor diet, low physical activity, prolonged stress, and intrinsic factors, such as mitochondrial dysfunction leading to reduced adenosine 5′-triphosphate (ATP) production, inflammation, and hypoxia (106, 117). Oxidative stress affects both the structure and function of the skin. It leads to changes in the structure of skin matrix proteins, such as collagen and elastin, and consequently, to loss of skin elasticity and firmness, the formation of wrinkles, and pigmentation disorders (116–118). Oxidative stress is also responsible for impaired immune responses in the skin (119). In the context of inflammatory skin diseases, oxidative stress can disrupt the skin's hydrolipid barrier, damage keratinocyte DNA, oxidize lipids in the stratum corneum, and induce the production of pro-inflammatory cytokines—interleukin-1β (IL-1β) and interleukin 33 (IL-33)—in the dermis, promoting inflammation and exacerbating the disease (120).

**Figure 3:**
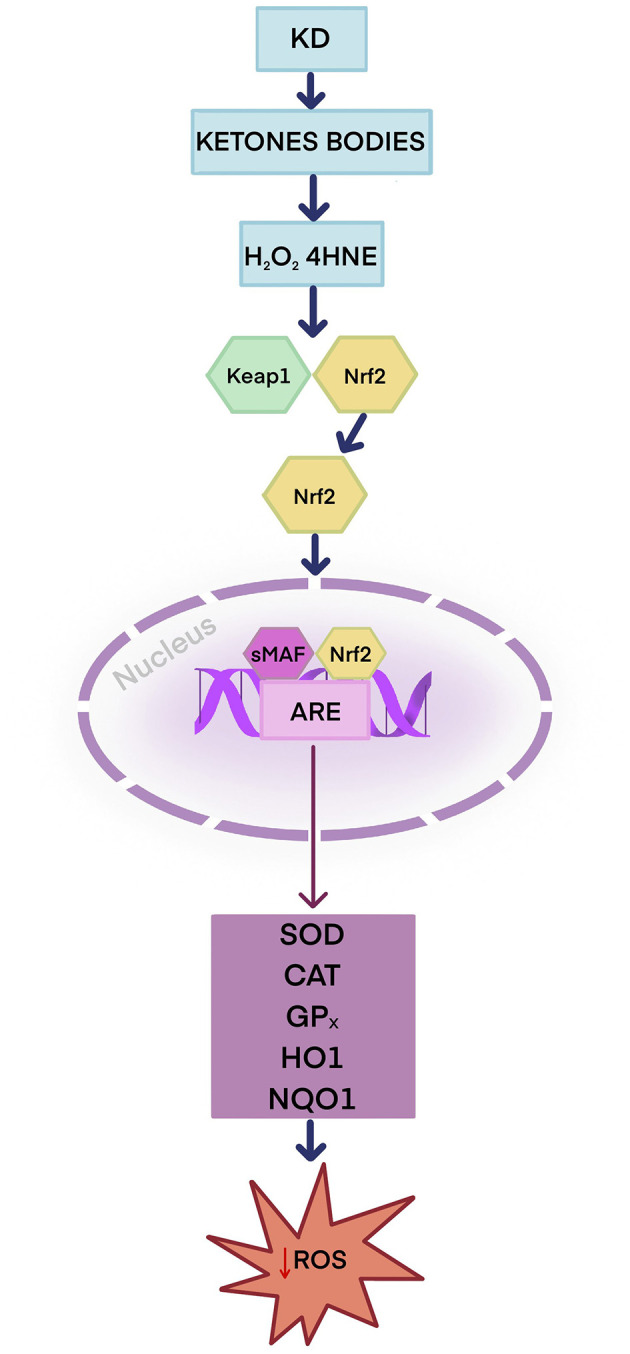
Potential antioxidant role of the ketogenic diet—Keap1–Nrf2–ARE antioxidant response pathway. The ketogenic diet produces ketone bodies that enhance the production of H_2_O_2_ and 4-HNE. H_2_O_2_ and 4-HNE activate Nrf2. Nrf2 is released from the Nrf2–Keap1 complex and travels to the cell nucleus, where it forms heterodimers with sMAF proteins, allowing it to bind to ARE sequences in DNA. Nrf2 activates antioxidant and detoxification enzymes: SOD, CAT, GP_X_, HO-1, and NQO1. ARE, antioxidant response element; CAT, catalase; GP_X_, glutathione peroxidase; 4-HNE, 4-hydroxynonenal; H_2_O_2_, Hydrogen peroxide; HO-1, heme oxygenase; KD, ketogenic diet; Keap 1, Kelch-like ECH-associated protein 1; Nrf2, nuclear factor erythroid 2-related factor 2; NQO1, NAD(P)H quinone dehydrogenase 1; SOD, superoxide dismutase; sMAF, small musculoaponeurotic fibrosarcoma protein.

As an exogenous source of antioxidants, the diet plays a significant role in protecting against oxidative stress. Dietary antioxidants neutralize reactive oxygen species, maintain redox balance, and prevent skin disease (121). Current literature suggests that KD supports the body's ability to combat oxidative stress by producing ketone bodies, which may alleviate the symptoms of many skin conditions linked to oxidative stress (14). There are two potential mechanisms by which KD may enhance the body's natural antioxidant capacity. The first mechanism is that ketones stimulate nuclear factor erythroid 2-related factor 2 (Nrf2) (14, 19, 122). Nrf2 plays a key role in protecting cells from oxidative stress-induced damage (14, 19). Under physiologic conditions, Nrf2 is present in the cytosol in an inactive form as a complex with the inhibitory protein Keap1. Keap1 plays a vital role in regulating the ubiquitination of Nrf2 and, at a later stage, its degradation (19, 123). An animal study showed that rats on a ketogenic diet during metabolic adaptation (approximately 3 weeks) exhibited increased production of H_2_O_2_ originating from the hippocampus mitochondria and 4-hydroxynonenal (4-HNE), a product of lipid peroxidation. These reactive molecules have a signaling function, modifying the Keap1 protein, which leads to Nrf2 activation (124). Nrf2 is released from the Nrf2–Keap1 system and then translocates to the cell nucleus, where it combines with small musculoaponeurotic fibrosarcoma (sMAF) proteins to form heterodimers. The Nrf2–sMAF heterodimer binds to the antioxidant response element (ARE), which leads to the transcription of genes encoding antioxidant and detoxification enzymes such as SOD, CAT, GP_X_, heme oxygenase 1 (HO-1), NAD(P)H quinone dehydrogenase 1 (NQO1), which protect cells from oxidative stress and toxins (19, 124–126) (Figure 3).

By influencing metabolic pathways, KD can also indirectly modulate NAD^+^/NADH levels, which play an important role in maintaining redox homeostasis (19, 127). NAD^+^ is an electron carrier in glycolysis and the Krebs cycle, where it is converted into the reduced form NADH, which is essential for ATP production (127, 128). NAD^+^ also plays an important role in DNA repair, serving as a substrate for poly-(ADP-ribose) polymerase (PARP) enzymes that are involved in the response to DNA damage (127–130). PARP, and PARP-1 in particular, uses NAD^+^ to add ADP-ribose residues and target proteins to DNA (127–129). This modification leads to the remodeling of chromatin structure and the recruitment of DNA repair proteins, such as X-ray repair cross-complementing protein 1 (XRCC1), DNA ligases, and DNA polymerases (129, 130). In addition, NAD^+^ activates sirtuins (127–130). Sirtuins represent a family of seven NAD^+^-dependent enzymes (SIRT1–SIRT7) (129). Sirtuins, particularly SIRT1 and SIRT3, protect cells from oxidative stress by increasing the activity of antioxidant enzymes, including superoxide dismutase 2 (SOD2) and CAT (127, 131, 132). In addition, SIRT1 reduces oxidative stress and inflammation by inhibiting the nuclear factor kappa B (NF-κB) signaling pathway, resulting in decreased production of inflammatory cytokines, including IL-1β, interleukin-6 (IL-6), tumor necrosis factor (TNF-α), and ROS (133–135). In contrast, SIRT3 and SIRT5 improve mitochondrial function, as manifested by reduced ROS production during oxidative phosphorylation (136). With age, NAD^+^ levels decrease while NADH levels increase, leading to metabolic disorders, mitochondrial dysfunction, oxidative stress, and accelerated aging (137). A disrupted NAD^+^/NADH balance may contribute to the development of many diseases, including cardiovascular and neurodegenerative disorders such as Alzheimer's and Parkinson's disease, metabolic disorders such as type 2 diabetes, and inflammatory skin conditions such as acne and psoriasis (135–137). It has been reported that the NAD^+^/NADH ratio is more important for maintaining good health than NAD^+^ alone (138, 139). KD may increase the NAD^+^/NADH ratio through various mechanisms. Firstly, restricted carbohydrate intake in KD leads to a reduction in glycolysis, the primary pathway of glucose catabolism, which is responsible for the production of NADH (140). Restriction of glycolysis results in reduced NADH production, which may contribute to an increase in the NAD^+^/NADH ratio (141, 142). By inhibiting glycolysis, KD reduces oxidative stress due to decreased ROS production (122, 143). Secondly, ketone bodies are metabolized in mitochondria, resulting in less NAD^+^ consumption and an increase in the NAD^+^/NADH ratio in the cytoplasm (144). However, further studies are needed to clarify the exact mechanism by which KD affects NAD^+^/NADH levels. Current knowledge is largely derived from preclinical studies investigating the biological processes involved, with a lack of clinical trials confirming these effects in human subjects.

## 6 The ketogenic diet and the gut microbiota

The gut microbiota, also known as the gut microbiome, is a collection of diverse species of bacteria, fungi, and protozoa that colonize the human gastrointestinal tract from birth (145). It is estimated that the gut microbiota comprises about 100 trillion microorganisms (145). These bacteria are part of the *Firmicutes, Bacteroidetes, Actinobacteria, Proteobacteria, Fusobacteria*, and *Verrucomicrobia* families, with *Firmicutes and Bacteroidetes* being predominant (145–147). A qualitative or quantitative disturbance of the microbiota colonizing the gut is known as dysbiosis (146–148). Dysbiosis impairs the intestinal barrier, increasing its permeability and allowing bacteria and lipopolysaccharides (LPS) from the gut to translocate into the systemic circulation, leading to systemic inflammation (148). The gut microbiota plays an important role in the function of the immune system and metabolic activity (148–150). It is responsible for the breakdown of complex and undigested polysaccharides, particularly mucin, resistant starch, and oligosaccharides, which the human body cannot metabolize on its own (151, 152). Subsequently, the gut microbiota produces short-chain fatty acids (SCFAs), such as butyrate, acetate, and propionate, which affect intestinal homeostasis (147, 153). It also plays a key role in the synthesis of vitamins, including vitamins K and B12 (153).

Studies indicate that the type of delivery significantly impacts the composition of newborns' gut microbiota (154). It has been found that the gut microbiota of babies born naturally is rich in *Bifidobacteria, Lactobacillus*, and *Bacteroides* (155). In contrast, babies delivered by cesarean section have poorer gut microbiota, dominated by *Staphylococcus, Streptococcus*, and *Clostridium*, and a significant deficiency of *Bifidobacteria* (155). It is believed that the first 2 years of life are crucial for the development of the immune system and the formation of the gut microbiome (156). Various factors, including lifestyle, diet, stress, antibiotics, and bacterial infections, can also modulate the gut microbiota (147, 157). Among these, diet has the most significant impact on the gut microbiota (157). Dietary interventions can modulate both the composition and function of the gut microbiota (157). Poor dietary habits, particularly a diet high in saturated fats and refined carbohydrates, lead to changes in the composition of the gut microbiome. This increases the *Firmicutes*/*Bacteroidetes* ratio, which is associated with obesity (158, 159). In contrast, foods rich in dietary fiber increase the diversity of the gut microbiota and the production of metabolites, including SCFAs, which have anti-inflammatory effects (160, 161).

The literature highlights an important relationship between the gut microbiota and the skin, referred to as the gut–skin axis (162). A growing body of evidence suggests that many skin diseases, such as acne vulgaris, psoriasis, atopic dermatitis, and HS, may result from an imbalance of the gut microbiota (162). Nevertheless, these studies often report varied and inconsistent findings (162). Several studies in patients with psoriasis and psoriatic arthritis have observed an increased abundance of *Firmicutes* in the gut microbiota (162). *Firmicutes* are Gram-positive bacteria involved in fat production and storage (162). An increased ratio of *Firmicutes* to *Bacteroidetes* results in a weakened intestinal mucosal barrier, which promotes the passage of bacteria into the bloodstream and leads to chronic inflammation. This is due to a change in carbohydrate metabolism, specifically an increased production of the pro-inflammatory acetate and a decreased production of butyrate, which strengthens the intestinal barrier and has anti-inflammatory effects (162). Recent reports indicate that acne may also result from changes in the gut microbiota (21, 162–164). However, studies on the *Firmicutes/Bacteroidetes* ratio in acne are inconclusive. Two authors demonstrated a reduced *Firmicutes/Bacteroidetes* ratio in acne patients (163, 164). Also shown was an increase in *Proteobacteria*, whose significant numbers damage the intestinal barrier, enhancing inflammation. Another study found a high *Firmicutes/Bacteroidetes* ratio in acne patients before antibiotic therapy (164). Discordant research findings may be attributed to varied research methodologies and characteristics of the control group, including differences in age, sex, and socioeconomic factors. Further research is needed to evaluate the impact of the gut microbiota on inflammatory skin diseases.

KD may modulate the gut microbiota, which may be vital in some inflammatory skin diseases. Several studies indicated that KD increases the *Bacteroidetes/Firmicutes* ratio (165, 166). *Bacteroidetes* increase lipid metabolism, which is associated with fat loss (165). Obesity is a known risk factor for some inflammatory skin conditions, such as psoriasis and HS (167, 168). By increasing *Bacteroidetes*, KD may lead to weight loss and alleviate the symptoms of the above diseases (162, 165). *Bacteroidetes* also produce SCFAs, which may reduce systemic inflammation typical of inflammatory skin diseases (162). SCFAs, especially butyrate, modulate the immune system by activating G-protein-coupled receptors (GPCRs), particularly G-protein-coupled receptor 43 (GPR43) and G-protein-coupled receptor 109A (GPR109A) (169). GPCRs promote the differentiation of regulatory T cells (Tregs) by activating mTOR and inhibiting the histone deacetylase (HDAC) pathway (169). Tregs are responsible for inhibiting immune responses by reducing the severity of chronic inflammation. SCFAs inhibit the NF–κB pathway and reduce the production of pro-inflammatory cytokines such as IL-1β, IL-6, and TNF-α, which can alleviate skin symptoms in psoriasis, acne, or HS (169). Another study found that KD also reduces the amount of *Proteobacteria*, which are linked to inflammation (170). Although KD may potentially modulate the composition of the gut microbiota, current clinical evidence in dermatological patients is minimal, and the majority of the data come from studies conducted in individuals with obesity or epilepsy. Further well-designed randomized controlled trials in dermatological populations are needed to assess the long-term impact of this intervention on skin diseases.

## 7 Ketogenic diet in inflammatory skin diseases

### 7.1 Acne

Acne vulgaris is one of the most common chronic inflammatory skin diseases. It is estimated to affect approximately 9.4% of the world population (171, 172). Acne predominantly appears during adolescence, but is also increasingly common in adulthood (173). The clinical manifestations of acne are comedonal, papular, nodular, and cystic lesions, which can result in scarring, post-inflammatory hyperpigmentation, or, less commonly, hypopigmentation (172). Acne vulgaris lesions most commonly affect the face, neck, back, and chest (172). A significant seborrhea is also observed in acne (172).

The etiopathogenesis of acne is complex and multifactorial. It is most commonly associated with increased sebum production in the sebaceous glands, abnormal hyperkeratinization of the hair follicles and their excessive colonization by *C. acnes*, and the release of inflammatory mediators in the skin (172–174). Other causes include genetic predisposition, hormonal factors, poor skin care, and even diet (172). The skin condition of acne patients has also been shown to be significantly dependent on the gut microbiota (175). The gut microbiota plays an important role in the pathophysiology of acne vulgaris, potentially via interaction with the mTOR pathway (21, 174). Increased mTOR activity conditions excessive keratinocyte proliferation and increased lipogenesis in sebocytes, ultimately leading to sebum overproduction (21). Metabolites derived from the gut microbiota regulate various metabolic functions, such as proliferation and lipid metabolism, which are mediated by the mTOR pathway (21). The mTOR pathway regulates the intestinal barrier, which can, in turn, influence the composition of the intestinal microbiota (21). Excessive activation of mTOR can also lead to increased intestinal permeability and dysbiosis (21). Recent data indicate that oxidative stress also significantly impacts the course of acne vulgaris (21, 176, 177). Overproduction of ROS is involved in the pathogenesis of acne vulgaris via various pathways, including activation of mTOR, peroxisome proliferator-activated receptors (PPARs), and toll-like receptors (TLRs) (21, 176–178). ROS induce inflammation by increasing the production of pro-inflammatory cytokines, especially IL-1, IL-8, and TNF-α (176, 177). NOD-like receptor pyrin domain-containing protein 3 (NLRP3) may be involved in the pathogenesis of acne vulgaris (179). Studies have shown that the NLRP3 inflammasome is also involved in other skin diseases, such as psoriasis, urticaria, and bullous pemphigoid (179). The leading acne-associated bacterium, *C. acnes*, stimulates the immune system through NLRP3 activation (180). Subsequently, NLRP3 leads to the activation of caspase 1. This enzyme mediates the inflammatory response by producing pro-inflammatory cytokines, such as IL-1β and IL-18, which contribute to the development of acne vulgaris (180).

The importance of diet in the course and treatment of acne vulgaris is also increasingly emphasized; however, research findings are still limited and inconclusive. Recent reports indicate that high blood insulin levels exacerbate acne (21). Foods with a high glycemic index (GI) cause a sharp rise in glucose levels, leading to a sudden surge in insulin release (181). Elevated insulin levels cause an increase in insulin-like growth factor (IGF-1) and a decrease in insulin-like growth factor-binding protein 3 (IGFBP-3) (19–21). This results in excessive keratinocyte and sebocyte proliferation, as well as decreased activity of the retinoic acid receptor (RAR) and retinoid X receptor (RXR) in the skin, causing increased comedogenicity and higher sebum production (19, 182). In addition, increased levels of insulin and IGF-1 reduce the synthesis of sex hormone-binding globulin (SHBG) (19). SHBG is a protein produced in the liver that binds to testosterone, dihydrotestosterone (DHT), and estradiol (183). Low SHBG levels cause an increase in free testosterone (fT) in the blood, which is converted into DHT by the enzyme 5-α-reductase (184). DHT is responsible for increased sebum production by the sebaceous glands and exacerbates acne lesions (21, 185, 186).

**Figure 4:**
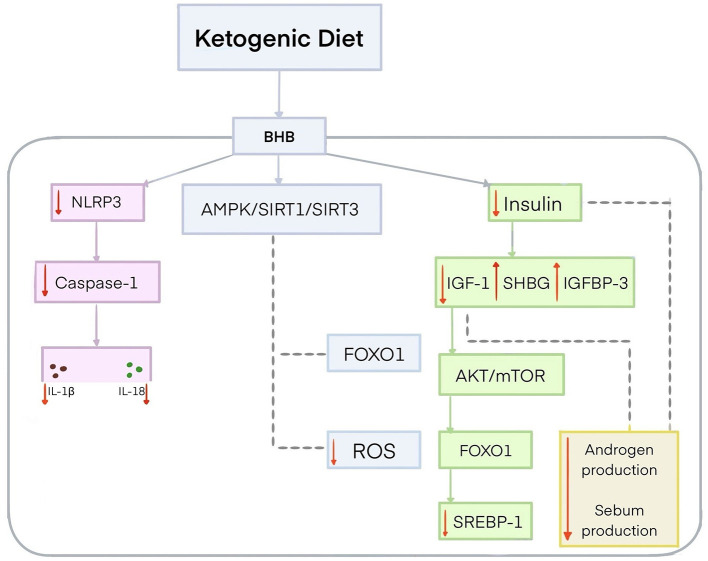
Potential mechanism for the effect of the ketogenic diet on acne. The ketogenic diet produces BHB, which inhibits the activity of NLRP3 and thereby reduces the pro-inflammatory cytokines IL-1β and IL-18 by inhibiting caspase-1 activation. BHB activates AMPK/SIRT1/SIRT3 signaling, leading to FOXO1 inhibition and reduced ROS levels. BHB contributes to lower insulin levels, leading to a reduction in IGF-1 and an increase in IGFBP-3. The reduction of insulin and IGF-1 increases SHBG concentrations, resulting in reduced androgen activity and lower sebum production. Decreased IGF-1 concentrations lead to AKT/mTOR inhibition and FOXO1 activation. FOXO1 inhibits SREBP1. AKT, protein kinase B; AMPK, adenosine 5′-monophosphate-activated protein kinase; FOXO1, forkhead box protein O1; IGF-1, insulin-like growth factor; IGFBP-3, insulin-like growth factor-binding protein 3; IL-1β, interleukin-1β; IL-18, interleukin 18; mTOR, mechanistic target of rapamycin; NLRP3, NOD-like receptor pyrin domain-containing protein 3; SHGB, sex hormone-binding globulin; SIRT 1, sirtuin 1; SIRT 3, sirtuin 3; SREBP1, sterol regulatory element-binding transcription factor 1.

The effect of KD on acne is a growing area of research. It has been speculated that KD may alleviate symptoms of acne vulgaris and even completely suppress them due to its anti-inflammatory and antioxidant properties (21). Ketone bodies exert their anti-inflammatory effects through different mechanisms. BHB is the leading and most biologically active ketone body produced during KD use (21, 187). It plays a significant role in promoting moderate mitochondrial stress, which activates Nrf2, adenosine 5′-monophosphate-activated protein kinase (AMPK), and SIRT1 and SIRT3, all of which are involved in anti-inflammatory and antioxidant responses (21, 188). This activation may further contribute to the inhibition of forkhead box protein O1 (FOXO1) and the reduction of ROS (189). In addition, BHB inhibits NLRP3 activation, resulting in a reduced release of inflammatory factors, particularly IL-1β and IL-18, by inhibiting caspase-1 activation (21). Several studies have shown that the ketogenic diet, especially VLEKT, can reduce acne lesions by lowering insulin levels (21, 176, 190). This contradicts the Western pattern diet, which stimulates insulin secretion with only a slight increase in blood glucose levels (21, 176). KD lowers insulin, causing a decrease in IGF-1, which consequently leads to an increase in IGFBP-3 (21). Furthermore, a decrease in insulin and IGF-1 levels is associated with an increase in SHGB (21). An increase in SHGB reduces free testosterone and sebum production (21, 191). A decrease in IGF-1 leads to reduced activity of the protein kinase B (AKT)/mTOR pathway, which in turn activates FOXO1 (21, 192). FOXO1 inhibits sterol regulatory element-binding transcription factor 1 (SREBP1) (193). SREBP1 plays an important role in lipid metabolism. A reduction in SREBP1 activity results in less sebum production and reduced acne lesions (Figure 4) (194). A study evaluating the effectiveness of a 45-day VLEKT in improving acne in 31 young women with moderate acne and grade I obesity showed a reduction in markers of oxidative stress, such as reactive oxygen metabolite derivatives (dROMs), and a decrease in pro-inflammatory metabolites, including trimethylamine N-oxide (TMAO). The study indicates that the VLEKT may be a potential therapeutic tool for treating skin diseases associated with oxidative stress and inflammation (176). However, further research is needed to evaluate the interaction mechanism between ketones and NLRP3 and determine its efficacy and safety as a treatment option for acne vulgaris (13, 19, 182). There is still no reliable evidence confirming VLEKT's effectiveness in treating acne vulgaris. Existing studies are mostly preliminary, involve small cohorts, and lack long-term randomized controlled trials, which limits the strength of current conclusions.

### 7.2 Psoriasis

Psoriasis is a chronic, non-infectious, systemic inflammatory disease of autoimmune origin. It affects approximately 2–3% of the world's population (195). According to available data, it is most commonly observed in Caucasians and Scandinavians, while Asian and African populations are less affected (196). Psoriasis affects both men and women, although it usually develops earlier in women (197, 198). It can develop at any age, but is most common in people aged 15–30 (199). Typical sites of psoriatic lesions include the scalp, elbows, knees, hands, feet, and the lumbosacral region (197, 200).

The typical symptoms of psoriasis are skin lesions resulting from epidermal hyperproliferation, increased angiogenesis, and accumulation of inflammatory cells (201). However, the clinical symptoms of psoriasis may vary and depend on its type. The most common is plaque psoriasis (202, 203). The classic clinical manifestations of plaque psoriasis are well-demarcated, erythematous, scaly plaques covered with silvery scales (202, 203). Another form is pustular psoriasis, characterized by numerous pustular lesions filled with pus (202). Pustular psoriasis has two variants—generalized and localized palmoplantar (202). The most severe variant of psoriasis is the generalized form, which is often accompanied by systemic symptoms (202).

The pathogenesis of psoriasis is multifactorial and is still not fully understood. Immunological factors play a prominent role in the development of this disease (204). Psoriasis involves uncontrolled activation of T helper cells (Th1) and (Th17), resulting in the overproduction of pro-inflammatory cytokines, such as IL-1, IL-6, IL-23, IL-22, interferon alpha (IFN-α), and TNF-α (204–206). Genetic factors also play a key role in the pathogenesis of psoriasis (204, 207). The human leukocyte antigen (*HLA-Cw6*) is considered one of the strongest alleles determining the development of this disease (199, 207). It affects the activation of T cells, especially CD8+ T cells, which then recognize *HLA-Cw6* antigen presentation (208). In turn, T cells produce inflammatory cytokines, such as IL-17 and IL-23, which exacerbate skin inflammation (206, 209). The presence of *HLA-Cw6* affects the course and severity of psoriasis, as well as comorbidities. Some studies indicate that *HLA-Cw6* in patients with psoriasis may be associated with better biologic treatment efficacy, particularly with IL-23 and IL-17 inhibitors (207). Environmental factors also contribute to the development of psoriasis (195, 199). These include a history of infection, the use of certain drugs, especially TNF-α inhibitors, smoking, alcohol, and stress (195, 199).

The relationship between psoriasis and obesity is bidirectional. Chronic systemic inflammation is believed to be the key pathomechanism underlying this relationship (167, 201). Numerous studies have shown that obesity is a risk factor for psoriasis, affecting its course and the Psoriasis Area and Severity Index (PASI) (205, 210). Excess visceral fat leads to increased production of pro-inflammatory cytokines, including TNF-α, IL-6, and IL-17, as well as the following adipokines: leptin, visfatin, and resistin, which exacerbate inflammation (13, 205). A more severe course of this disease was found in obese patients compared to normal-weight patients (203, 205, 210). Obesity also negatively affects the efficacy of biologic treatment in patients with psoriasis (205). A second direction of the interrelationship between psoriasis and obesity indicates that patients with psoriasis have elevated levels of inflammatory cytokines, particularly TNF-α, which can lead to metabolic abnormalities, including insulin resistance, and may consequently lead to the development of obesity (211). Clinical trial meta-analyses have shown that weight loss resulting from dietary interventions and increased physical activity in patients with psoriasis significantly reduces the severity of disease symptoms (212, 213).

Diet plays a significant role in the course of psoriasis (13, 214, 215). Dietary factors can be an effective part of psoriasis therapy, helping to alleviate disease symptoms and modulating systemic inflammation and the gut microbiota (214). One study showed that KD and MD may benefit patients with psoriasis (205). KD's potential role in alleviating psoriasis symptoms may be due to several mechanisms. The first relates to weight reduction, which is important as obesity is one of the factors contributing to psoriasis exacerbation (201, 205). Another mechanism may be the anti-inflammatory effect of KD. KD reduces inflammatory cytokines, such as IL-6, IL-17, and IL-23, which have a significant role in the etiopathogenesis of psoriasis (201, 205). The last relates to the antioxidant effects of KD. It has been suggested that ketone bodies produced while on a KD may reduce ROS, which is associated with increased inflammation (205).

One clinical trial evaluated the effects of KD on obesity, psoriasis, and psoriatic arthritis (205). It included 26 patients who were randomly assigned a KD or MD for 8 weeks. This was followed by a 6-week washout period. After its completion, patients switched to the other diet for another 8 weeks. The study assessed various clinical and biochemical markers, including body weight, body mass index (BMI), waist circumference (WC), total fat mass, and visceral fat, as well as such inflammatory markers as the PASI and the Disease Activity Index of Psoriatic Arthritis (DAPSA). Both diets were found to have a beneficial effect on reducing body weight, total fat mass, visceral fat, BMI, and WC. With regard to these parameters, KD was found to be slightly more effective than MD. The study also showed that KD affected inflammatory markers. Patients at the start of the study had moderate psoriasis with a mean PASI of 5.09 ± 5.73. After KD treatment, the PASI was 3.15 ± 4.88. This diet significantly reduced the PASI score (*p* = 0.04) compared to the baseline. In contrast, the PASI score after MD did not change significantly (3.82 ± 3.93, *p* = 0.278 vs. baseline). The difference between diets was statistically significant (*p* = 0.038). Inflammatory cytokines, such as IL-6, IL-17, and IL-23, also decreased after KD. In contrast, MD showed no significant changes in inflammatory markers (205).

Another study evaluated the effect of VLEKT on patients with psoriasis (195). The study enrolled 30 patients aged 18–65 with obesity and plaque psoriasis. Study participants were on a VLEKT for 4 weeks. Clinical assessments covered body weight, BMI, waist and hip circumference, visceral fat, PASI, Dermatology Life Quality Index (DLQI), visual analog scale (VAS) for itch ratings, and measurement of the following inflammatory cytokines: IL-1β, IL-2, IL-4, interferon (IFN-γ), and TNF-α. VLEKT resulted in significant weight loss and improved PASI (by 50%), DLQI, and VAS scores for pruritus and pain. There was also an improvement in the levels of metabolites closely associated with psoriasis, particularly, an increase in folic acid, vitamin B12, calcium, and bilirubin. Reduced concentrations of inflammatory cytokines, including IL-2 and IL-1β, were also reported. The other cytokines tested—IL-4, TNF-α, and INF-γ—did not show any significant changes, probably due to the short study period. VLEKT also increased L-glutamine and glutamate levels, and decreased L-alanine, L-leucine, and choline. Changes in their levels may serve as potential biomarkers for the early diagnosis and prognosis of this disease. Although the findings of both studies are promising for treating psoriasis, they involved a limited number of participants and were conducted over a short period. Further studies with a larger patient population and evaluation of the long-term impact of the VLEKT on patients with psoriasis are required. At present, there is still no high-quality randomized controlled trial confirming the efficacy of VLEKT in psoriasis, which highlights the need for more robust clinical evidence (195).

### 7.3 Hidradenitis suppurativa

Hidradenitis suppurativa (HS), also known as acne inversa, is a chronic inflammatory disease mainly affecting areas with a high concentration of apocrine glands (216). The etiology of this disease is not fully understood. It is suggested that it may be caused by immune disorders, hormonal imbalances, genetic predisposition, obesity, smoking, and environmental factors, including diet (216). Inflammatory cytokines, such as IL-17 and TNF-α, are particularly important in the pathogenesis of HS (217). In addition, dysbiosis may also contribute to the development and clinical severity of HS (218). An imbalance of the gut microbiota can lead to immune activation and inflammation, particularly through an increase in pro-inflammatory lipopolysaccharides (LPS), which results in increased production of inflammatory cytokines, such as TNF-α, IL-1β, and IL-6 (218). Changes in the gut microbiome during HS can also lead to reduced production of anti-inflammatory SCFAs, which in turn inhibit pattern recognition receptors (PRRs) involved in pathogen recognition and reduce the production of IL-10 and TGF-β (218). Patients with more severe HS are also found to have elevated levels of TMAO, which is associated with inflammation and cardiometabolic risk (216, 219). TMAO may be a potential biomarker of disease progression (219). HS usually emerges in adolescence and is recurrent (216). It is much more common in women, especially those between the second and third decades of life (219). HS symptoms include painful nodules and abscesses that can lead to the development of fistulas and scarring (216).

Diet plays a significant role in the course and treatment of HS (216). Many studies indicate that specific foods can exacerbate or alleviate the symptoms of this disease (216, 220). Dietary factors exacerbating HS include dairy products, foods with a high glycemic index, and brewer's yeast (220). Obesity is considered one of the important risk factors for this disease, possibly through systemic inflammation (216). A correlation has been found between high BMI and HS symptom severity (216). The introduction of dietary modifications may lead to weight reduction and alleviate disease symptoms (220). Research suggests that VLEKT has therapeutic potential in treating HS symptoms, particularly through its anti-inflammatory effects, reduction of body fat, and changes in the gut microbiota composition, which modulate systemic inflammation and oxidative stress (216).

A pilot study evaluating the effect of a 28-day VLEKT on clinical HS severity in 12 overweight and obese women revealed weight loss and a reduction in HS severity as measured by the Sartorius score (216). The Sartorius score is a clinical tool designed to assess the severity of HS. It involves counting individual skin lesions, including fistulas, abscesses, nodules, and scars in seven anatomical regions. It also measures the greatest distance between two similar lesions in each anatomical region, such as axillae, buttocks, genitalia, and others, on both the left and right sides of the body (216). There was also a decrease in metabolic markers, including TMAO, which is associated with improved gut microbiota, oxidized low-density lipoprotein (OxLDL), and decreased oxidative stress, as evidenced by reduced dROM levels. Although VLEKT has therapeutic potential in reducing HS symptoms, further studies on a larger population and randomized controlled trials are necessary to confirm the effectiveness of VLEKT in improving HS symptoms. Existing evidence is limited to preliminary observations from small cohorts and short follow-up, which does not allow for firm clinical conclusions (216).

## 8 The ketogenic diet and skin cancer

Recent literature indicates that KD may be a promising adjuvant therapy for many malignant tumors. It has been shown that KD may have anti-tumor potential for several cancer types, including colorectal, brain, breast, and pancreatic cancer (39, 81, 82). The majority of the studies suggest that KD may counteract the Warburg effect (20, 82). The Warburg effect is a metabolic process in which tumor cells convert glucose to lactate, even in the presence of oxygen, which promotes tumor progression (20, 82). The potential mechanism of KD is to reduce the availability of glucose to tumor cells, depriving them of energy, inhibiting their proliferation, and tumor growth (13, 20, 82). Recent reports indicate that the reverse Warburg effect may also occur in the tumor formation process, whereby tumor cells undergo oxidative phosphorylation (OXPHOS) instead of glycolysis (221). However, the reverse Warburg effect does not apply to all tumors (221). Recent evidence suggests that KD may also be beneficial in skin cancers, particularly melanocyte-derived melanoma (222).

Cutaneous melanoma is a rapidly growing malignant skin tumor with a propensity to metastasize (223). It develops from neuroectodermal melanocytes (224). Melanocytes are located in the basal layer of the epidermis and synthesize melanin, which plays a key role in photoprotection against UV radiation (224). It has the highest mortality rate compared to other skin tumors, such as basal cell carcinoma and cutaneous squamous cell carcinoma (223, 224). Cutaneous melanoma at an advanced stage is resistant to standard cancer treatment (225). UV radiation is considered one of the most important inducers of cutaneous melanoma (223, 224). Other causes include a genetic predisposition, a high number of pigmented nevi, and a fair complexion (223, 224).

The literature emphasizes the potential impact of dietary choices on the risk of developing cutaneous melanoma (225). A clinical control study conducted on a group of 273 patients with cutaneous melanoma and a control group of 269 healthy individuals evaluated the impact of an anti-inflammatory diet on the risk of developing cutaneous melanoma (225). A study shows that participants who followed an anti-inflammatory diet, incorporating at least eight anti-inflammatory food products, had a lower risk of melanoma. The odds ratio (OR) for the association between an inflammatory diet and melanoma risk was 0.29 with a 95% confidence interval (CI) of 0.17–0.49. The study also included genetic analysis, which focused mainly on the −765 G > C polymorphism in the cyclooxygenase-2 (*COX2*) gene. COX2 is an enzyme that plays an important role in the synthesis of prostaglandins involved in inflammatory responses (225). The regulation of COX2 expression is complex, involving transcriptional and post-transcriptional mechanisms influenced by cytokines, growth factors, and pro-inflammatory stimuli (225). Overexpression of COX2 is reported in many types of cancer, including cutaneous melanoma, which contributes to tumor progression and angiogenesis (225). It was found that this polymorphism did not increase the risk of melanoma (225).

One *in vivo* study assessed the effect of KD on the progression and metastatic development of cutaneous melanoma (222). The study was conducted in a mouse model with BRAF and NRAS mutations, as well as wild-type melanoma xenografts and allografts of highly metastatic melanoma (222). The *BRAF gene* is located on chromosome band 7q34. This gene encodes the serine/threonine kinase B-Raf. BRAF plays a vital role in the mitogen-activated protein kinases/extracellular signal-regulated kinases (MAPK/ERK) signaling pathway, which influences cell growth and proliferation (226). The *BRAF mutation*, in particular the valine to glutamic acid mutation (V600E), is associated with many types of cancer, especially cutaneous melanoma. It leads to the activation of the MAPK pathway, which conditions the uncontrolled proliferation of tumor cells (226). The *NRAS gene* encodes the N-ras protein (227). NRAS mutations in melanoma occur mainly at codon 61, with Q61R considered the most common (227). They activate the RAS/MAPK pathway, which promotes cell proliferation and survival (227). A study on the effect of KD on the skin used immunocompetent (mutation-free) and immunocompromised mice (BRAF V600E, NRAS Q61R) (222). KD was shown to slow tumor growth in immunocompromised mice that had genetically and metabolically differentiated melanoma xenografts, indicating that it can inhibit tumor progression regardless of the genetic basis of the tumors. Reductions in metastasis were also found in immunocompetent mice with highly metastatic melanoma allografts, indicating that KD may not only slow tumor growth but also reduce the translocation of tumor cells to other sites. KD has also been shown to cause changes in amino acid metabolism, in particular, a reduction in levels of α-aminoadipic acid, a key biomarker of cancer (222). These changes were also observed in tumor xenografts. KD also contributed to increased sphingomyelin levels and enhanced hydroxylation of sphingomyelins and acylcarnitines, which determine changes in cell membrane composition. The increase in sphingomyelins can interfere with signaling pathways, including phosphatidylinositol 3-kinase (PI3K)/AKT and MAPK, inhibiting cancer cell proliferation and growth (222).

The role of KD as an adjuvant therapy for cutaneous melanoma appears promising (222). However, current evidence is mainly derived from preclinical studies, and further investigations, particularly rigorous clinical trials in humans, are necessary to clarify the mechanisms involved and to evaluate their efficacy and safety in patients with cutaneous melanoma.

## 9 Systemic complications of KD

The initial stage of KD is often accompanied by short-lived symptoms, commonly referred to as “keto flu,” including fatigue, gastrointestinal disturbances, and headache, which usually resolve within a few days (228, 229). Patients may also experience a characteristic breath odor due to acetone formation (228, 230).

Studies indicate that KD can be used in both short and long terms (48). However, long-term use may be associated with several complications, the most common of which is nutrient deficiency (231). Nutritional deficiency disorders can impair the immune system, increasing susceptibility to infection and impairing wound healing (232).

Another serious complication associated with the use of KD involves potential changes in the lipid profile, particularly an increase in low-density lipoprotein (LDL) cholesterol (48). Long-term use of KD may also increase the risk of kidney stones (231, 233). The incidence of nephrolithiasis in patients on KD ranges from 3 to 10% (233).

## 10 Adverse effects of the ketogenic diet on the skin

### 10.1 Prurigo pigmentosa

The benefits of KD for the skin are promising, but with its increasing popularity, prurigo pigmentosa (PP) cases are being reported more frequently (234). PP is a rare inflammatory skin disease (234). This disease was first described in 1971 by Nagashima, a Japanese dermatologist (234, 235). The majority of the cases have been reported in East Asia (234). The disease is also believed to have affected other ethnic groups, but was misdiagnosed due to the doctors' ignorance (236, 237). Today, PP is diagnosed in patients worldwide (234, 238). It affects women much more often than men (234, 239). Typically, the disease emerges in adolescents or young adults (239). Its clinical manifestations include itchy, erythematous papules and patches, and sometimes vesicles arranged in a reticular pattern (234, 235). After the papules resolve, post-inflammatory hyperpigmentation emerges (234, 235). The histopathological picture of PP at an early stage shows a perivascular neutrophilic infiltrate (236, 239). At a later stage, spongiosis and ballooning of keratinocytes are observed (236, 239). A lymphocytic infiltrate and melanophages follow this in the papillary layer of the dermis (235, 238, 239). PP is a recurrent disease (235). The lesions typically develop on the neck, chest, and back (235). The etiology of PP is not fully explained (234, 238). PP has been linked to Still's disease, pregnancy, chafing from clothing, excessive sweating, or *Helicobacter pylori* (236–238). In addition, it is believed that KD, fasting, untreated diabetes, anorexia nervosa, and bariatric surgery may also induce PP, resulting from increased production of ketone bodies in the liver (236, 237). Some patients with PP have been found to have high levels of ketone bodies in both urine and blood (239). Accumulation of ketone bodies in the blood vessels induces neutrophilic inflammation (239). Several case studies have shown a relationship between KD and PP (Table 3).

**Table 3:** Characteristics of patients with prurigo pigmentosa induced by the KD.

**Sex/age**	**Origin**	**Location of lesions**	**PP emergence after KD**	**Treatment**	**Treatment outcome**	**Reference**
M/21	Hispanic	Chest and back	2 months	Oral doxycycline 100 mg twice a day, KD discontinuation	2 weeks	(234)
F/26	N/A	Chest, back, supraclavicular region	3 weeks	KD discontinuation	1 month	(262)
F/26	Saudi Arabia	Chest, back	3 months	Oral doxycycline 100 mg/d, KD discontinuation	2 weeks	(238)
M/20	Saudi Arabia	Neck	2 months	Oral doxycycline 100 mg/d, KD discontinuation	2 weeks	(238)
F/22	Saudi Arabia	Chest, neck	4 months	Oral doxycycline 100 mg/d, KD discontinuation	2 weeks	(238)
F/21	N/A	Chest, inframammary region, neck	1 week	KD discontinuation	2 months	(263)
M/24	Caucasian	Neck, chest, back	9 days	KD discontinuation, topical corticosteroids	Several days	(264)
M/16	N/A	Trunk, proximal limbs	1 week	KD discontinuation, topical corticosteroids	15 days	(239)
F/16	Denmark	Back, armpits, abdomen	2 weeks	Oral tetracycline 500 mg twice a day, topical Betnovate with chinoform, KD discontinuation	1 month	(265)
M/18	Denmark	Chest, abdomen, back	2 weeks	Oral tetracycline 500 mg twice a day, topical Betnovate with chinoform, KD discontinuation	1 month	(265)

F, female; M, male; N/A, not applicable; KD, ketogenic diet.

In the majority of the cases described, doxycycline 100 mg/day was used to treat PP. However, tetracycline, minocycline, dapsone, and topical corticosteroids are also mentioned as treatment options (234, 235, 237). KD discontinuation and a return to a balanced diet are also necessary. Some patients did not use drug treatment, and PP lesions resolved after KD discontinuation (234). Further studies are needed to clarify the exact mechanisms by which KD influences the development of PP.

### 10.2 Nutritional deficiencies and skin manifestations in ketogenic interventions

Nutritional deficiencies also negatively affect the appearance of the skin, often causing a number of severe skin conditions (240). In addition to altering appearance, such deficiencies can weaken skin barrier function and resilience (240). Studies indicate that long-term use of KD conditions vitamin C, biotin, and zinc deficiencies (20).

Vitamin C is an important antioxidant that plays a key role in collagen synthesis (241, 242). Its deficiency may lead to impaired wound healing, accelerated skin aging, and, in severe cases, scurvy (241, 243). Several cases of scurvy in children due to KD use have been reported in the literature (64, 244). Until recently, scurvy was considered a historical and forgotten disease; however, recent case reports significantly challenge this assumption (64). Typically, symptoms of scurvy emerge after 1–3 months of significant vitamin C deficiency (245, 246). These include systemic symptoms, such as fatigue, a deteriorated mood, and muscle and joint pain (246, 247). Bleeding gums, anemia, and loss of appetite are also observed (246). Scurvy also affects the skin, particularly causing excessive keratinization of hair follicles, bruising, bloody spots on the skin, and impaired wound healing (245, 246). In a reported case of a 12-year-old girl with cerebral palsy, cystic encephalomalacia, and refractory epilepsy, scurvy was confirmed after long-term KD use (244). The clinical signs were knee swelling, anemia, and bleeding gums. The patient showed intolerance to ready-made ketogenic products, so her primary source of nutrition was blenderized KD in a 2:1 ratio. The patient's KD contained several ingredients, including avocado, chicken breast, double cream, and coconut oil. The diet was supplemented with iodized salt, calcium carbonate, fish oil, and a multivitamin/mineral supplement (Phlexy-vits, Nutricia). The applied KD was shown to provide her with 60–70% of the recommended dietary allowance (RDA) for vitamin C (45 mg/day). Vitamin C was administered at a dose of 500 mg/day for 1 month during the initial stage of scurvy treatment. The dose was then reduced to 250 mg/day. Vitamin C supplementation led to a rapid improvement in symptoms (244). Another report found a case of scurvy following KD use in a 5-year-old girl on the autism spectrum (64). The patient presented with periorbital edema, multiple ecchymotic patches, bleeding gums, poor appetite, weight loss, generalized weakness, and hypochromic anemia. Laboratory tests showed severe vitamin C deficiency. Treatment included supplementation with vitamin C, multivitamins, and elemental iron. The supplementation resulted in a marked improvement (64).

Biotin (vitamin B7) supports keratin production and is important for skin, hair, and nail health (248). Its deficiency, although rare, may present with dermatitis, brittle nails, and hair loss (248). One study assessed the effect of KD on biotin levels using an animal model (249). The study was conducted on 32 male mice divided into four groups: a control (C) group receiving a standard diet with biotin, a biotin-deficient (BD) group on a biotin-free diet, a ketogenic control (KC) group on a ketogenic diet (low-carbohydrate and high-fat) with biotin, and a ketogenic biotin-deficient (KBD) group on a ketogenic diet without biotin. The key comparison was between the KC group (KD with biotin) and the KBD group (KD without biotin), which allowed the researchers to assess whether KD alone increases biotin requirements or accelerates the development of biotin deficiency. The study lasted 9 weeks, during which clinical signs associated with biotin deficiency and biochemical parameters, such as serum and tissue biotin levels (liver, kidney, brain, testes), and biotin-dependent enzyme activity of 3-methylcrotonyl CoA carboxylase (MCC), propionyl-CoA carboxylase (PCC), pyruvate carboxylase (PC) and acetyl-CoA carboxylase (ACC), and blood glucose levels, were evaluated. After 5 weeks, mice in the KBD group showed symptoms of biotin deficiency, including hair loss and dermatitis. The KBD group also had the lowest biotin and fasting glucose levels, likely due to impaired gluconeogenesis. In addition, the expression of MCC, PCC, PC, and AC decreased, indicating metabolic dysfunction (249). Although the results suggest that KD exacerbates biotin deficiency in mice, human clinical studies are needed to confirm these observations (249).

Zinc plays an important role in collagen synthesis and wound healing (250). Its deficiency may lead to skin lesions, impaired wound healing, and increased hair loss (243, 251). Severe zinc deficiency is also associated with acrodermatitis enteropathica, a rare autosomal recessive genetic disorder (252). Acrodermatitis enteropathica causes impaired intestinal absorption of zinc (252). Acquired acrodermatitis enteropathica is also known to be caused by insufficient dietary zinc intake (253). A distinctive sign of the disease is dermatitis, which presents as well-defined erythematous and eczematous plaques, erosions, scabs, and even blisters (254, 255). Diarrhea, conjunctivitis, alopecia, and photophobia are also observed (254, 256). To date, only one case of acrodermatitis enteropathica has been reported among patients using KD—it emerged in an 11-year-old boy with drug-resistant epilepsy (253). The patient presented with mild alopecia and perioral, periorbital, and perigenital eruption. Due to swallowing problems, the patient used a liquid KD formulation containing amino acids and medium- and long-chain triglycerides for 3 months. Despite medical recommendations, the parents did not provide the child with vitamin and mineral supplements. Laboratory tests showed a significant zinc deficiency (15 mcg/dL) compared to the normal range (50.0–120.0 mcg/dL). In turn, biopsy of skin lesions showed superficial epidermal necrosis with dyskeratotic keratinocytes. Treatment included discontinuing KD, administering zinc gluconate 50 mg/day, and emollients. Following treatment, symptoms improved after three days and completely resolved within a month (253).

An appropriately composed KD can minimize side effects (257). Although KD significantly limits carbohydrate intake, it maintains the minimum carbohydrate supply necessary for gluconeogenesis (48). A key element of KD is the avoidance of highly processed foods. Vegetable oils, particularly olive oil, coconut oil, rice oil, MCTs, and avocado oil, are the best sources of fats (48, 257). Brazil nuts, macadamia nuts, walnuts, hazelnuts, and almonds, which are rich in essential fatty acids (EFAs), are also particularly recommended as part of KD (257). Fatty fish, such as mackerel, salmon, eel, and sardines, also contain healthy fats (257). Meat is an equally important part of KD, as a key source of protein and fat. Pork, beef, and poultry are good choices (257). Eggs are also a particularly recommended animal product (257). Dairy products used in KD include full-fat cheeses (Gorgonzola, Mascarpone, Cheddar, Brie, Robiola), full-fat yogurts without added sugar, cream, and butter (48, 257). KD also relies heavily on vegetables, particularly cruciferous and leafy green vegetables, tomatoes, aubergines, broccoli, and mushrooms (48). Recommended fruits include avocados, strawberries, lemons, blueberries, raspberries, coconuts, and olives (48). Permitted beverages on KD are water, tea, and coffee (258). Popular KD meals include omelets, scrambled eggs, salads, beef steaks, and chicken with vegetables (48). The food industry also offers a wide range of ready-made ketogenic products, such as biscuits, focaccia, crackers, and desserts, which vary in nutritional composition (257). Liquid KD formulas with the classic 4:1 ketogenic ratio (4 g of fat per 1 g of protein and carbohydrates combined) are also available (257). These are usually enriched with essential fatty acids, dietary fiber, and vitamins. They are recommended for infants, children, and adults. Another food product for special medical purposes is a powder ketogenic formula for preparing a liquid mixture (257). The formula has a high-fat content with a ketogenic ratio of 4:1 or 3:1 and is fortified with a vitamin and mineral complex, essential fatty acids, and sometimes MCTs (257). For infants, when dissolved in water, this product provides a complete meal tailored to their nutritional needs. Studies indicate that a powder ketogenic formula is an effective, safe, and well-tolerated adjuvant therapy for drug-resistant epilepsy in pediatric patients (257).

## 11 Study limitations and future prospects

The research findings to date on the effects of KD on the skin indicate potential benefits. However, they have several important limitations. The majority of the available studies include only short-term observations (a few weeks), making it impossible to assess the long-term effects of KD on the skin. Another crucial methodological shortcoming is the small size of the study groups (*n* < 50). The limited sample size significantly hinders the interpretation of the results. It may result in increased statistical errors due to low test power. A major limitation of many studies investigating the effect of KD on the skin is the lack of randomization, which significantly reduces their scientific value. The lack of standardization in the ketogenic protocols used in research on skin conditions is also a considerable impediment to the synthesis of results. The macronutrient ratios used in studies vary, which makes it difficult to compare their effects on skin processes. Despite promising reports on KD's effects on the skin, a key direction for future research should be to clarify the specific molecular mechanisms and signaling pathways in different layers and cells of the skin. At the same time, randomized clinical trials are needed to precisely determine the optimal timing of dietary intervention and standardize the dietary protocol for macronutrient ratios and calorie intake. They should also include an analysis of potential interactions with conventional treatments and an assessment of KD's long-term safety in skin conditions. A key practical limitation is the difficulty of maintaining long-term adherence to ketogenic interventions, which may reduce their feasibility and sustainability in dermatological practice. Moreover, nutritional deficiencies associated with prolonged KD use (e.g., vitamin C, biotin, and zinc) can impair skin integrity, highlighting the importance of careful monitoring. Importantly, future studies should investigate whether responses to different KD variants vary by sex or age, as these factors may influence both metabolic and dermatological outcomes. A further promising direction is the integration of nutrigenomic approaches to personalize ketogenic interventions, with the potential to optimize efficacy and minimize risks. Addressing these gaps will be essential for establishing evidence-based guidelines for the safe and practical application of ketogenic therapies in dermatology.

## 12 Summary

This review summarizes current knowledge on the effects of KD on the skin, particularly its potential therapeutic role in inflammatory skin diseases, such as acne vulgaris, psoriasis, hidradenitis suppurativa, and skin cancer. In addition, the impact of KD on the gut microbiota and its potential adverse effects, such as PP and nutrient deficiencies, were also presented. KD is characterized by a dietary pattern that predominates in fats, has a minimal amount of carbohydrates, and a moderate intake of protein. It has been successfully used to treat drug-resistant epilepsy in children for more than a century. KD is also finding applications in reducing obesity and treating insulin resistance, type 2 diabetes, neurodegenerative diseases, and cardiovascular disorders. There is evidence that this diet can also alleviate symptoms of inflammatory skin conditions through its anti-inflammatory and antioxidant effects, as well as modulation of the gut microbiota. The antioxidant potential of KD may be due to its impact on the Nrf2 pathway. Activation of the Nrf2 pathway leads to the induction of antioxidant and detoxifying enzymes, significantly reducing oxidative stress. KD can also reduce oxidative stress by increasing the NAD^+^/NADH ratio. It is speculated that KD's anti-inflammatory mechanism may be due to the reduction of blood insulin levels, inhibition of the NF–κB pathway, and NLRP3, which blocked the production of pro-inflammatory cytokines. Scientific evidence suggests that KD may alter the composition of the gut microbiota, reducing pro-inflammatory *Proteobacteria* and increasing the ratio of *Bacteroidetes* to *Firmicutes*, which could potentially be beneficial in inflammatory skin diseases. Recent reports indicate that KD may also be an adjuvant therapy for cutaneous melanoma. Despite the potential benefits of KD for skin health, randomized clinical trials are needed to evaluate its efficacy and long-term effects, as well as the exact mechanisms by which it affects the skin.
